# Graphene foam as a biocompatible scaffold for culturing human neurons

**DOI:** 10.1098/rsos.171364

**Published:** 2018-03-07

**Authors:** Giovanna M. D'Abaco, Cristiana Mattei, Babak Nasr, Emma J. Hudson, Abdullah J. Alshawaf, Gursharan Chana, Ian P. Everall, Bryony Nayagam, Mirella Dottori, Efstratios Skafidas

**Affiliations:** 1 Department of Biomedical Engineering, Melbourne School of Engineering, The University of Melbourne, Carlton, Victoria, Australia; 2 Centre for Neural Engineering, Melbourne School of Engineering, The University of Melbourne, Carlton, Victoria, Australia; 3 Department of Psychiatry, Royal Melbourne Hospital, The University of Melbourne, Carlton, Victoria, Australia; 4 ARC Centre of Excellence for Integrative Brain Function, The University of Melbourne, Carlton, Victoria 3010, Australia; 5 Department of Anatomy and Neuroscience, School of Biomedical Sciences, The University of Melbourne, Carlton, Victoria, Australia; 6 Department of Audiology and Speech Pathology, University of Melbourne and Bionics Institute Melbourne, Carlton, Victoria, Australia; 7 Illawarra Health and Medical Research Institute, University of Wollongong, Wollongong, New South Wales, Australia

**Keywords:** graphene foam, biocompatibility, human stem cells, human cortical neurons

## Abstract

In this study, we explore the use of electrically active graphene foam as a scaffold for the culture of human-derived neurons. Human embryonic stem cell (hESC)-derived cortical neurons fated as either glutamatergic or GABAergic neuronal phenotypes were cultured on graphene foam. We show that graphene foam is biocompatible for the culture of human neurons, capable of supporting cell viability and differentiation of hESC-derived cortical neurons. Based on the findings, we propose that graphene foam represents a suitable scaffold for engineering neuronal tissue and warrants further investigation as a model for understanding neuronal maturation, function and circuit formation.

## 1. Background

Effective repair of neurological injury will depend on the culture of precisely differentiated, mature neurons, and materials that provide advantages in this aim are of great interest. Among the many choices that are available, graphene-based materials are emerging as a promising candidate. The culture of neural stem cells (NSCs) derived from brain tissue (of human foetal and rodent foetal and adult origin) on graphene substrates has been reported. These studies demonstrate that graphene is biocompatible for the growth of NSCs, providing excellent attachment properties that then confer enhanced neuronal differentiation and maturation towards neural networks [1–8]. In addition to its excellent biocompatible nature, graphene is a biomaterial with conductive properties, a feature that positions this material as a standout candidate in the field of regenerative medicine [7,9]. Inducing neural progenitors to differentiate and fully mature into functional networks is a challenge that, if met, could pave the way for treatment opportunities of neurological disorders. In this regard, a conductive substrate such as graphene can be used to instruct developing neurons to mature and form networks. As graphene is shown to have superior biocompatibility and enhanced capacity to guide neuronal differentiation of human NSCs (hNSCs), this has paved the way for further studies whereby graphene has been used in various stimulation techniques including electrical stimulation to enhance neuronal commitment[1,10–14]. These studies clearly demonstrate that using such techniques it is possible to interface neurons with graphene and achieve a greater degree of neuronal maturation that supports the formation of functional circuits. In addition, there are examples of successful functionalization of graphene not only to enhance neuronal differentiation using hNSCs [15] but also to influence and direct mesenchymal stem cells towards neuro-induction and neural differentiation [16].

The cerebral cortex is mostly composed of glutamatergic excitatory and GABAergic inhibitory neurons both of which are critical in the development and regulation of neural networks [17]. For this reason, any disruption of such networks may represent an aetiological factor for neurological disorders. Therefore, materials that can aid the culture and maturation of human neurons will help to advance our knowledge of brain development and brain disease. The aforementioned studies highlight the advantages of the use of graphene as a substrate in the field of neural tissue engineering, leading us to assess the potential for its use in the culture of neurons derived from human embryonic stem cells (hESCs). Using hESCs, it is possible to specifically drive neural subtype specification [18]. In this model, we are able to derive neural epithelium tissue in a process termed neural induction and commitment towards neuronal differentiation [18]. By this process, hESCs can be differentiated towards dorsal (glutamatergic) and ventral (GABAergic) cortical forebrain neurons [19]. However, neuronal maturation and network formation is a prolonged process [20,21]. As graphene is known to provide a more stable environment for neuronal differentiation and maturation using NSCs, this led us to assess its biocompatibility for the culture of hESC-derived neurons.

A recent advance in the field of graphene scaffolds is the fabrication of graphene foam [22]. Graphene foam is particularly popular owing to the highly porous nature of its structure that offers two key advantages: (i) high surface/volume ratio, thereby supporting extended culture time course without affecting cell viability, permitting long-term longitudinal studies, and (ii) high porosity that can support the exchange of fresh nutrients and waste products. Graphene foam is known to be biocompatible using rodent neuronal cultures [2,23]. In these studies, graphene foam was shown to be capable of supporting growth and differentiation and circuit formation. In this study, we investigate the use of graphene foam as a potential basis for culturing hESC-derived cortical neurons which, to our knowledge, to date, has not been reported. We provide evidence of biocompatibility of both glutamatergic and GABAergic-fated neurons and show data illustrating that this scaffold is able to maintain viability and neuronal differentiation equally as well as the two-dimensional (2D) monolayer system.

## 2. Material and methods

### 2.1. Reagents

Growth factors: bFGF (recombinant human fibroblast growth factor-basic; PeproTech) and EGF (recombinant human epidermal growth factor; PeproTech), DMEM/F-12 medium (1 : 1) and Neural Basal medium (Gibco/Life Technologies), glucose at 30% (w/v) in distilled water (ThermoFisher Scientific) and stored at 4°C (d-(+)-glucose, Sigma-Aldrich, Australia), GlutaMAX™-1 (Gibco/Life Technologies), phosphate-buffered saline solution (PBS) (Sigma-Aldrich, Australia) made according to the manufacturer's instructions, poly-d-lysine hydrobromide (Sigma-Aldrich, Australia) reconstituted in double distilled water at 1 mg ml^−1^ and stored at 20°C, laminin supplied reconstituted at 1 mg ml^−1^ Tris–HCl (50 mM, pH 7.4), NaCl (0.15 M) and stored at 20°C (Invitrogen/Life Technologies), ITS solution (insulin/transferrin/selenium-A solution, Gibco/Life Technologies), N-2 supplement (Gibco/Life Technologies), B27 retinol-free supplement (Gibco/Life Technologies) and Pen/Strep solution (penicillin/streptomycin solution with 10 000 U ml^−1^ penicillin and 10 000 mg ml^−1^ streptomycin, Gibco/Life Technologies).

### 2.2. Human embryonic stem cell culture

The H9 cell line (WA-09, WiCell) was cultured using organ culture dishes (Centre Well Organ Culture Dish, FALCON Corning, Inc.) coated with vitronectin (Vitronectin XF™, STEMCELL Technologies) using mTeSR™1 defined medium (STEMCELL Technologies) and maintained at 37°C and 5% CO_2_. The coating of plates and medium preparation were according to the protocols provided by the manufacturer (STEMCELL Technologies). Every 7 days, colonies were mechanically dissected and transferred to freshly coated plates. Cell culture media were replenished daily.

### 2.3. Neural induction

Neural inductions were set up as previously established [24]. In brief, hESCs were mechanically dissected into pieces (approximately 0.5 mm in width) and then transferred to laminin-coated organ culture plates. For laminin-coating, plates were firstly coated with poly-d-lysine solution (10 µg ml^−1^ in PBS) and kept at room temperature for 30 min. This solution was aspirated and this was followed by the addition of laminin solution (10 µg ml^−1^ in PBS) and incubated overnight at 4°C. The next day, the laminin solution was removed and replaced with neural induction culture medium, termed N2B27. This medium consisted of a 1 : 1 mixture of neurobasal medium with DMEM/F12 medium supplemented with ITS 1%, N-2 1%, B27 2%, glucose 0.3%, GlutaMAX 1% and Pen/Strep 50 U ml^−1^/50 µg ml^−1^ for 14 days. For differentiation towards glutamatergic neurons, cultures were maintained in N2B27 supplemented with the small-molecule inhibitor SB431542 (10 µM, Tocris) and noggin (500 ng ml^−1^, R&D Systems) for 7 days followed by N2B27 supplemented bFGF (20 ng ml^−1^) for 7 days. For differentiation towards GABAergic neurons, the small-molecule smoothened agonist (SAG, 400 nM, Calbiochem/Merck) was also supplemented in the media together with SB431542 and noggin during the first week of neural induction, followed by the addition of bFGF (20 ng ml^−1^) a further 7 days [25,26].

### 2.4 Neurosphere cultures

 Following two weeks of neural induction, neural progenitors were dissected into pieces and transferred to individual wells of a 96-well ultra-low attachment cell culture plate (Costar/Corning, Inc., Corning, NY, USA), forming neurospheres. Neurospheres were cultured in neurosphere media (NSM) for a period of two weeks. NSM consists of neural basal media (NBM) supplemented with ITS 1%, N-2 1%, B27 2%, GlutaMAX 1%, Pen/Strep 50 U ml^−1^/50 µg ml^−1^, bFGF 20 ng ml^−1^ and EGF 20 ng ml^−1^ [27].

### 2.5. Set-up of two-dimensional cell culture

Two-week-old neurospheres were dissociated by gentle trituration (pipetting gently using a 200 µl Gilson pipette). These were then plated at a density of half a sphere/well in 24-well cell culture plates (Costar/Corning, Inc., Corning, NY, USA) that were previously coated with laminin (as described above) and cultured for a further 21 days in NSM in the absence of growth factor. For all immunofluorescence experiments, cells were cultured on 12 mm glass laminin-coated coverslips in 24-well cell culture plates.

### 2.6. Set-up of graphene for cell culture

Graphene foam was purchased from Graphene Laboratories, Inc. (Graphene Foam Calverton, NY, USA, dimensions 2 × 2 square inches, pore size 580 µm, thickness 200 µm). Graphene foam cell culture experiments were set up in 24-well cell culture plates. For setting up a graphene cell culture, graphene foam was prepared by cutting a circular piece measuring 12 mm (outer circumference). This was achieved by cutting the foam using a 12 mm biopsy punch (AcuPunch Acuderm, Inc., FL, USA). Next, using fine forceps, a 12 mm glass coverslip was placed into the cell culture well, followed by the graphene foam. The foam was held in place with an O-ring (15 mm in diameter (outer circumference) and 11 mm (internal circumference)), fabricated in acrylonitrile butadiene styrene using a three-dimensional (3D) printer. The graphene foam was then prepared for cell culture by laminin-coating the foam using the protocol described in §2.3. For all graphene cell culture set-ups, two-week-old neurospheres were used. These were harvested, dissociated as described above, counted, then plated at a density of 10^6^ cells well^−1^ and cultured for 21 days. At the conclusion of this time point, the graphene foam samples were processed for further experimental analysis.

### 2.7. Immunofluorescence

To process samples for immunofluorescence, samples were washed three times with PBS and then fixed with 4% paraformaldehyde for 15 min at room temperature. After washing three times in PBS, the samples were permeabilized in 0.2% Triton-X-100 in PBS for 15 min at room temperature and then blocked by incubation in 10% foetal calf serum in PBS for 60 min at room temperature. Samples were then incubated with primary antibody (diluted 1 : 500 in the block buffer) for 1 h at room temperature, washed 2–3 times in PBS followed by incubation with ALEXA-Fluor secondary antibody(s) (Life Technologies/Invitrogen) (diluted to 1 : 5000 in the block buffer) for 1 h at room temperature. After three washes in PBS, the samples were washed a final time in deionized water. Primary antibodies used: tubulinβ3 (mouse monoclonal, Chemicon MAB1637), MAP2AB (mouse monoclonal, Sigma-Aldrich, Australia, clone HM2), v-Glut (rabbit polyclonal, AbCam AB 77822) and GAD67 (mouse monoclonal, Millipore MAB5406). All samples were counterstained with 4’,6-diamidino-2-phenylindole (DAPI) (1 µg ml^−1^ final concentration) (Sigma-Aldrich, Australia). Samples grown in 2D (on glass coverslips) were mounted by inverting onto glass slides with 5 µl of moviol mountant. For the graphene samples, the foam was sitting on a glass coverslip. At the conclusion of the staining process, the foam sitting on the glass coverslip was mounted by inverting onto a glass slide with 5 µl of moviol mountant. Images were captured by epi-fluorescence using a ZEISS Observer z1 fluorescence microscope or confocal analysis using a ZEISS LSM 780 confocal laser scanning microscope with ZEN imaging software (Zeiss, Germany).

### 2.8. Scanning electron microscopy and helium ion microscopy

For imaging, the samples were washed with PBS and then fixed with 4% paraformaldehyde for 10–15 min at room temperature. This was followed by sequential dehydration with ethanol (30%, 50%, 75%, 85%, 95% and 100%: 15 min washing) followed by critical point drying. For scanning electron microscopy (SEM) imaging, the samples were sputter-coated with 5 nm of gold. SEM images were captured using a FEI Nova dual beam electron microscope (operating voltage 5 kV). Helium ion microscopy (HIM) images were captured using the Zeiss Orion NanoFab helium ion (operating/accelerating voltage is 30 kV). Image resolution of the microscope is specified to be approximately 2–3 Å with a working distance of 9 mm. The beam current was approximately 0.2 pA with a tilt angle of 30°. The samples were prepared as above for SEM imaging but not coated with any conductive material because any accumulated charges could be easily suppressed by the electron beam flood gun.

### 2.9. Chemical analysis

Chemical analysis was performed with the SEM (Philips XL30 scanning electron microscope) using energy-dispersive X-ray spectroscopy (EDX; Oxford Instruments) to provide further insights into chemical composition.

### 2.10. Raman measurements

Raman spectra are collected in ambient air and at room temperature with a DXRxi Raman imaging microscope (Thermo Scientific). An excitation laser with a wavelength of 532 nm and a mapped area of 100 × 100 µm^2^ are used. An incident power of 10 mW is used to avoid sample damage or laser-induced heating.

### 2.11. Quantitative real-time PCR

To assess expression profile differences, RNA was extracted from: (i) hESC, (ii) hESC-derived neurons grown for three weeks in 2D (monolayer), and (iii) hESC-derived neurons grown on the graphene foam for three weeks. Total RNA was extracted using the PureLink RNA Mini Kit (Life Technologies) according to the manufacturer's instructions. One microgram of RNA was used to synthesize first-strand cDNA with random primers using the SensiFAST cDNA Synthesis Kit (Bioline). House-keeping and specific probes were purchased from Life Technologies. Raw cycle threshold (*C*_t_) values for our candidate genes of interest were normalized for the endogenous controls GAPDH (house-keeping high expression: Hs02758991_g1), HMBS (house-keeping medium expression: Hs00609297_m1) and ELF1 (house-keeping low expression: Hs00152844_m1). Specific probes used in the analysis were: KI67: Hs01032443_m1, Caspase-3: Hs00234387_m1, tubulinβ3: Hs00801390_s1 and MAP2AB: Hs00258900_m1. Each reaction was run using 4.5 µl of cDNA template in a final reaction volume of 10 µl. Expression analysis was normalized against expression levels in the hESC sample. Gene expression was calculated using the $-2^{\Delta \Delta C_{t}}$ method [28]. Real-time PCR was performed with TaqMan Universal Master Mix (Applied Biosystems). Cycling parameters were set as the standard for TaqMan gene expression assays: 50°C for 2 min, 95°C for 10 min, then 40–45 cycles of 95°C for 15 s and 60°C for 1 min.

### 2.12. Statistical analysis: quantitative real-time PCR

For Q-RT-PCR analysis, statistical analysis was performed as follows. Each dataset represents the ±standard deviation (SD). The SD was calculated from *n* = 3 biological replicates and, for each biological sample, there were *n* = 3 technical replicates. The mean of the technical replicates/biological sample was calculated and used to calculate the SD for the biological replicates. Statistical significance was evaluated using the Student's *t*-test and significance was determined using the Holm–Sidak method with *α* = 5%.

## 3. Results

### 3.1. hESC-derived neurons engrafted onto graphene foam

**Figure 1.**
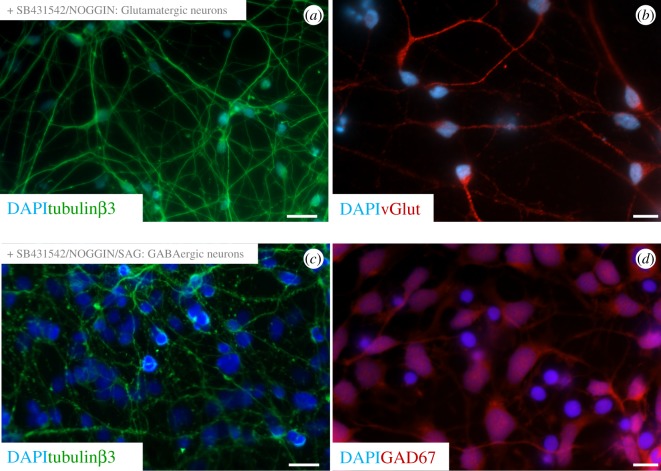
Derivation of glutamatergic and GABAergic cortical neurons from hESCs. Fluorescence images showing characteristic features of hESC-derived cortical neurons. Glutamatergic neurons characterized by immunostaining with (*a*) tubulinβ3 and (*b*) v-Glut. GABAergic neurons characterized by immunostaining with (*c*) tubulinβ3 and (*d*) GAD67. All samples counterstained with DAPI. Scale bars: (*a*,*c*) 20 µm, (*b*,*d*) 10 µm.

The protocol described by Denham & Dottori [24] and Chambers *et al*. [25,26] was employed to derive glutamatergic and GABAergic cortical neurons. The neuronal identity of both cell populations was then determined using immunofluorescence staining. To begin with, we assessed the expression of tubulinβ3. Tubulin is a component of the cytoskeletal and is the major constituent of the microtubule network. Expression of tubulinβ3 is restricted to neuronal populations and, as such, used routinely as a neuron-specific marker. Following neural commitment, expression of tubulinβ3 was detected in our neuronal cultures (figure 1*a*,*c*). The glutamatergic nature of neuronal fating was confirmed by the expression of v-Glut (vesicular glutamate transporter), which plays a vital role in the uptake of glutamate into synaptic vesicles at presynaptic nerve terminals of excitatory neurons. Following neural commitment/glutamatergic fating, expression of v-Glut was detected in the neuronal cell cultures (figure 1*b*). We confirmed the GABAergic nature of neuronal fating by examining expression of GAD67. The glutamic acid decarboxylase (GAD) proteins are responsible for the synthesis of GABA. There are two isoforms GAD65 and GAD67, and expression of the 67-kDa isoform (GAD67) is restricted to GABAergic neurons of the cortex. Following neural commitment/GABAergic fating, expression of GAD67 was detected in the neuronal cell cultures (figure 1*d*).

**Figure 2.**
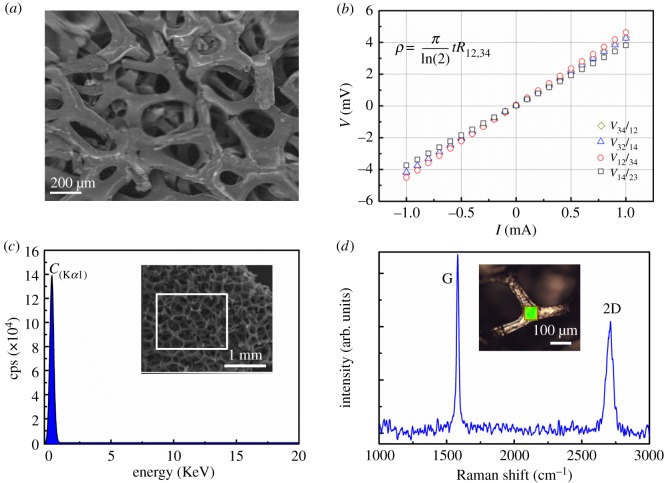
Graphene foam is an electroconductive substrate. (*a*) SEM image of graphene foam. Scale bar, 200 µm. (*b*) Electrical characterization of the graphene foam. A squared graphene foam with four connections (probes) at the corners labelled as 1, 2, 3 and 4 were prepared. The measurement was performed by applying current through 1 → 2 (*I*_12_), 2 → 3 (*I*_23_), 3→4 (*I*_34_), 1 → 4 (*I*_14_), and the voltage measured at the connections 3–4 (*V*_34_), 4–1 (*V*_41_), 1–2 (*V*_12_) and 2–3 (*V*_23_) correspondingly, which are plotted as the *V*–*I* viewgraph. Using the correction factor for thin samples and following the equation shown in the above figure, the conductivity and sheet resistivity were calculated where *t* is the thickness of foam (approximately 1 mm), *ρ*, the electrical resistivity; *V*, the potential difference; *I*, the current. (*c*) EDX spectrum of the graphene foam showing the C-K*α* line. (*d*) The G and 2D Raman bands of graphene foam.

Graphene foam is a highly porous, conductive structure (figure 2*a*,*b*) already known to be suitable for the culture of rodent-derived neurons [2]. The porosity enables the exchange of fresh nutrients and waste products during extended culture times. Conductivity would permit both electrical stimulation and electrical recording enabling the study of neuronal functionality and network formation. The conductive properties of the commercially supplied graphene foam were investigated and observed to follow Ohm's law (figure 2*b*). The van der Pauw method was used to measure the conductivity of the foams by taking the ratio between the electrical current (*I*) and voltage (*V*) [29]. The sheet resistance of the graphene foam was measured to be 16 Ω sq^–1^ and the conductivity was evaluated to be 0.5 S cm^–1^. The EDX spectroscopy of the graphene foam has also been performed as an additional characterization tool to confirm the phase purity. In the EDX spectrum, the C-K*α* line is clearly seen and no other spurious element is observed as shown in figure 2*c*. Furthermore, Raman spectroscopy was carried out under excitation laser (*λ *= 532 nm) on the graphene foam to provide complementary knowledge on chemical structure. Raman spectrum of graphene foam (figure 2*d*) reveals that the graphene material exhibits well-known G and 2D peaks approximately at 1581 and 2710 cm^−1^, which is consistent with the literature [30–32]. The calculated intensity ratio of 2D to G peaks, I2D/IG, is approximately 0.63 and less than one which implies that the graphene foam is made of multilayer graphene flakes.

**Figure 3.**
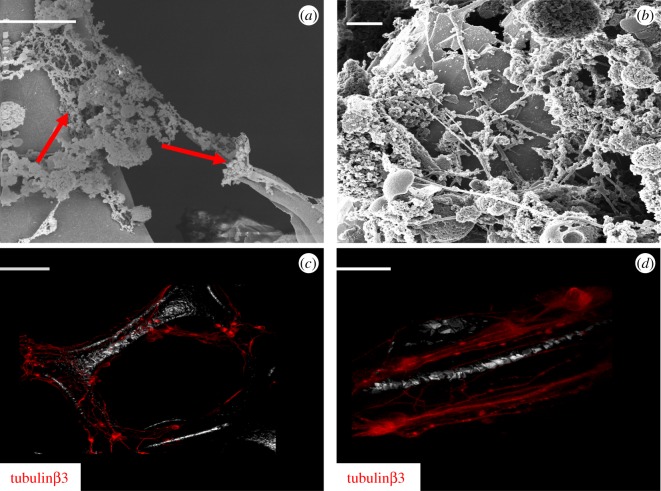
hESC-derived glutamatergic neurons can engraft onto graphene foam. (*a*) SEM image (scale bar, 10 µm). (*b*) HIM image (scale bar, 2 µm) of glutamatergic-fated neurons cultured on graphene foam. (*c*,*d*) Confocal images of glutamatergic-fated neurons cultured on graphene foam. Images show tubulinβ3 immunostaining. Scale bar: (*c*) 20 µm and (*d*) 10 µm. The white/greyscale image observed in the background shows the reflection of the scaffold.

**Figure 4.**
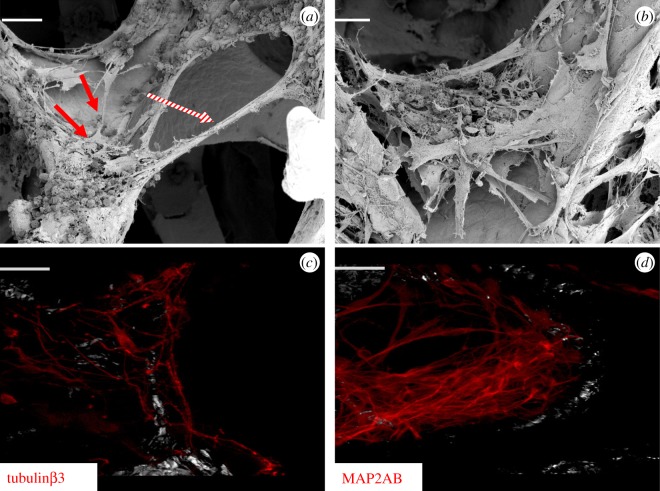
hESC-derived GABAergic neurons can engraft onto graphene foam. (*a*,*b*) SEM images of GABAergic-fated neurons cultured on graphene foam. Scale bar: (*a*) 50 µm and (*b*) 20 µm. (*c*,*d*) Confocal images of GABAergic-fated neurons cultured on graphene foam show positive expression of tubulinβ3 (*c*) and MAP2AB (*d*). Scale bar, 20 µm. The white/greyscale image observed in the background shows the reflection of the scaffold.

To assess the biocompatibility of graphene foams, neurospheres derived from either the glutamatergic or GABAergic neural induction protocols were mechanically disaggregated and cultured onto laminin-coated graphene foam for 21 days. Following this culture period, graphene foam scaffolds with either glutamatergic or GABAergic-fated neurons were subjected to SEM, HIM and immunofluorescence imaging (figures 3 and 4, respectively). Image analysis of glutamatergic-fated neurons showed that neurons engrafted onto the scaffold are capable of attaching and extending neurites along the fibres (figure 3*a*; solid red arrows), although we did not observe a capacity to grow and extend neurites across the pores of the scaffold. Glutamatergic neurons were additionally imaged using HIM imaging which provides superior contrast and resolution because of its increased depth of field (figure 3*b*). HIM imaging confirms that the neurons were capable of attaching to the scaffold and could extend complex neurite outgrowths along the scaffold. To confirm this, samples were examined using immunofluorescence staining with the neuronal marker, tubulinβ3, followed by confocal imaging (figure 3*c*,*d*). Both images show a similar pattern of attachment as observed using SEM and HIM.

SEM imaging of GABAergic-fated neurons showed that like glutamatergic neurons, GABAergic neurons also engraft onto the scaffold; however, we observed some differences in attachment (figure 4). Like the glutamatergic-fated neurons, GABAergic-fated neurons could form extensive neurite outgrowths along the graphene scaffold (figure 4*a*; solid red arrow) and were also capable of extending complex neurite outgrowth processes across the pores of the scaffold (figure 4*a*; hatched red arrow). Additionally, in contrast to glutamatergic neurons, GABAergic neurons were capable of forming 3D clusters within the porous cavities of the lattice (figure 4*b*). To confirm this, samples were also prepared for immunofluorescence analysis by staining with two cytoskeletal neuronal markers, tubulinβ3 and MAP2AB. Samples were assessed by confocal imaging and both images showed a similar pattern of attachment to that observed using SEM (figure 4*c*,*d*).

### 3.2. Viability and differentiation of hESC engraft onto graphene foam

**Figure 5.**
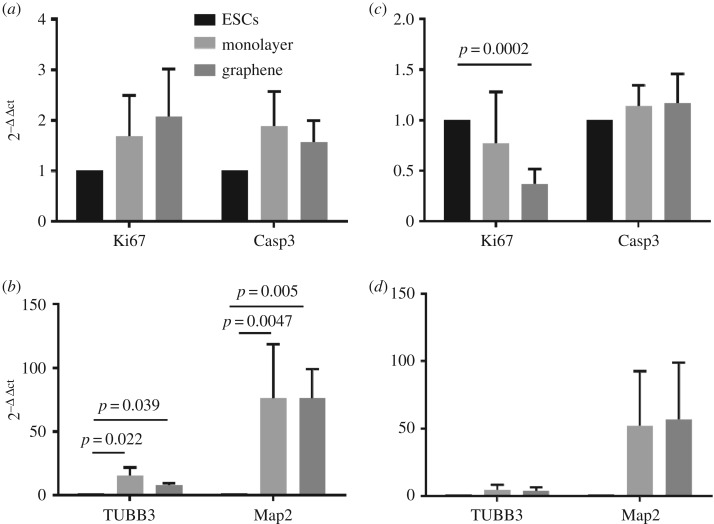
Graphene foam supports the culture of hESC-derived cortical neurons. (*a*,*c*) Expression analysis of Ki67 and Caspase-3 demonstrating that graphene is compatible with viability of hESC-derived neurons. (*a*) Results for glutamatergic neurons and (*c*) results for GABAergic neurons. (*b*,*d*) Expression analysis of neuronal maturation markers, tubulinβ3 and MAP2AB, demonstrating that graphene foam sustains neuronal differentiation. (*b*) Results for glutamatergic neurons and (*d*) results for GABAergic neurons. ESCs, hESCs; monolayer, neuronal cultures grown in 2D; graphene, neuronal cultures grown on the scaffold.

Having established capacity to engraft onto the scaffold, next we assessed biocompatibility by comparing the viability and neuronal maturation of cells cultured as a 2D monolayer with cells cultured on the scaffold (figure 5*a–d*). To achieve this, two-week-old neurospheres derived from glutamatergic and GABAergic neural inductions were gently dissociated and plated either as a monolayer onto laminin-coated dishes (2D) or onto a laminin-coated graphene foam. Following three weeks in culture, the biocompatibility characteristics of neurons cultured in 2D and in the foam were compared using Q-RT-PCR analysis. Cell viability was examined by assessing the expression levels of Ki67 (a marker of proliferation) and Caspase-3 (a marker of cell apoptosis) (figure 5*a*,*c*). Neuronal maturation was examined by assessing the expression of tubulinβ3 (TUBB3) and MAP2AB (Map2) (figure 5*b*,*d*).

Cell viability results obtained for glutamatergic neurons showed comparable expression levels of Ki67 and Caspase-3 between the 2D (monolayer) and the graphene foam culture conditions. This demonstrates that graphene foam supports the culture of glutamatergic cortical neurons equally as well as the 2D monolayer (figure 5*a*). The analysis of GABAergic neurons revealed a slightly different pattern of expression, in that expression levels of Ki67 were lower when cultured on the foam (figure 5*c*). This was not matched by increased expression of Caspase-3, which was equivalent across both the 2D and foam samples. We propose that the lower expression of Ki67 may indicate progression towards post-mitotic and more mature neuronal populations. Finally, we examined expression levels of tubulinβ3 and MAP2AB (figure 5*b*,*d*). Analysis of these markers in 2D monolayer and graphene foam cultures did not reveal any marked differences in expression levels of these neuronal markers. This suggests that, for the 21-day culture period, graphene is equally supportive of neuronal differentiation as the 2D cell culture model for both the glutamatergic and GABAergic-fated neurons (figure 5*b*,*d*).

## 4. Discussion

The culture of NSCs on graphene has shown that this scaffold can improve neuronal maturation and enhance functionality and circuit formation [7,9]. In addition, there are studies demonstrating that both chemical modification and stimulation can be used successfully in order to create micro-environments that can further improve control of stem cell fating towards neuronal commitment and network formation [7]. Graphene foam is attracting much attention as a scaffold for the culture of neurons. This interest stems from the fact that graphene is a conductive surface and being able to grow neurons on such an interface could permit the study of neuronal functionality, network formation and connectivity. Biocompatibility of graphene foam has been reported using cortical neurons derived from rodent tissue [2,23]. The first study reports the culture of post-natal day 1 mouse hippocampal-derived NSCs on graphene foam, wherein this group reports biocompatibility using a cell culture model consisting predominantly of neuronal and glial populations cultured for a period of two weeks in total [2]. In a subsequent study using post-natal day 2–3 rat hippocampal neurons, graphene foam was also observed to be biocompatible and, moreover, supportive of the assembly of neural networks that are more representative of the native physiological environment [23].

In this study, we investigated the use of graphene foam as a potential basis for culturing hESC-derived cortical neuronal cells, to date not reported. To achieve this aim, we adopted a well-characterized method protocol that directs hESC neural specification towards tissue that is enriched for forebrain neuronal populations [25]. This protocol together with protocols derived by our own laboratory provides capacity to channel neuronal fate towards either glutamatergic (excitatory) neurons or GABAergic (inhibitory) neurons [24,27]. Each population of human cortical neurons was cultured on graphene for a period of three weeks. At the end of this cell culture period, we examined cell viability and differentiation status. Our data demonstrated that graphene foam is a biocompatible scaffold capable of supporting cell viability and maintaining differentiation status of both types of hESC-derived cortical neurons. Our results show that our substrate is biocompatible and does not interfere with long-term survival of cultures.

The key features of graphene foam that facilitate such long-term cell culture are its porosity, which facilitates the efficient exchange of nutrients and high surface/volume ratio that provides the landscape. A major objective in the study of neuronal homeostasis is to advance our understanding of the mechanism underlying neuronal differentiation, maturation, functionality and underlying electrical connectivity. The ability to conduct long-term culture studies would allow human-derived cortical neurons to differentiate over the extended periods that are required to reach a more mature differentiation status. This will be crucial for functional studies and the analysis of neural network formation, which are known to establish over lengthy periods of time (greater than 100 days) [21,33,34]. As graphene foam is conductive in nature, this opens the door to electrical studies, making it possible to track neuronal circuit formation as the cells are maturing and acquiring function. In addition, based on the knowledge from the aforementioned studies that capitalize on functionalization and electrical stimulation [7], it could be possible to design a scaffold that guides and promotes neuronal maturation. Indeed, it may be possible to develop a platform that could promote faster maturation to the benefit of neural repair.

## 5. Conclusion

If our understanding of brain development and neurological disorders is to advance, the need to accurately model neuronal maturation and functionality is a priority. In this study, we demonstrate that it is possible to culture hESC-derived neurons on graphene foam. Our study shows that hESC-derived neurons can engraft onto the scaffold, showing minimal toxic effect for the first 21 days of cell culture, and is supportive of neuronal differentiation. The conductive nature of graphene foam means such a scaffold could be used to study neuronal connectivity and functionality. Our data provide the rationale for moving graphene foam forwards as a substrate for such studies. Additionally, it may be possible to establish hESC- and graphene-based organotypic models to study brain development that could represent better translational models for neuroscience discovery.

## Data accessibility

This article does not contain any additional data.
